# Management of Chyle Leak after Head and Neck Surgery: Review of Current Treatment Strategies

**DOI:** 10.1155/2017/8362874

**Published:** 2017-01-19

**Authors:** Sean W. Delaney, Haoran Shi, Alireza Shokrani, Uttam K. Sinha

**Affiliations:** ^1^Department of Otolaryngology Head and Neck Surgery, Keck School of Medicine, University of Southern California, 1540 Alcazar St, Suite 204Q, Los Angeles, CA 90033, USA; ^2^Department of Biochemistry and Molecular Biology, University of Southern California, Los Angeles, CA, USA

## Abstract

Chyle leak formation is an uncommon but serious sequela of head and neck surgery when the thoracic duct is inadvertently injured, particularly with the resection of malignancy low in the neck. The thoracic duct is the primary structure that returns lymph and chyle from the entire left and right lower half of the body. Chyle extravasation can result in delayed wound healing, dehydration, malnutrition, electrolyte disturbances, and immunosuppression. Prompt identification and treatment of a chyle leak are essential for optimal surgical outcome. In this article we will review the current treatment options for iatrogenic cervical chyle leaks.

## 1. Introduction

Chyle leak (CL) from iatrogenic thoracic duct injury is a rare but serious complication of head and neck surgery that occurs in 0.5–1.4% of thyroidectomies [1–4] and 2–8% of neck dissections [5–8]. The variable anatomy and fragile composition of the thoracic duct render it prone to inadvertent injury. The majority of CL transpires with surgery of the left neck; however, up to 25% of CL occur with right neck surgery [7, 8]. Although uncommon, CL would surely be encountered in any head and neck surgery practice. Early identification and appropriate management of a CL are imperative for optimal surgical outcome. In this article we aim to review the current treatment strategies for iatrogenic cervical chyle leaks.

## 2. Anatomy and Physiology of the Thoracic Duct

### 2.1. Embryology

**Figure 1:**
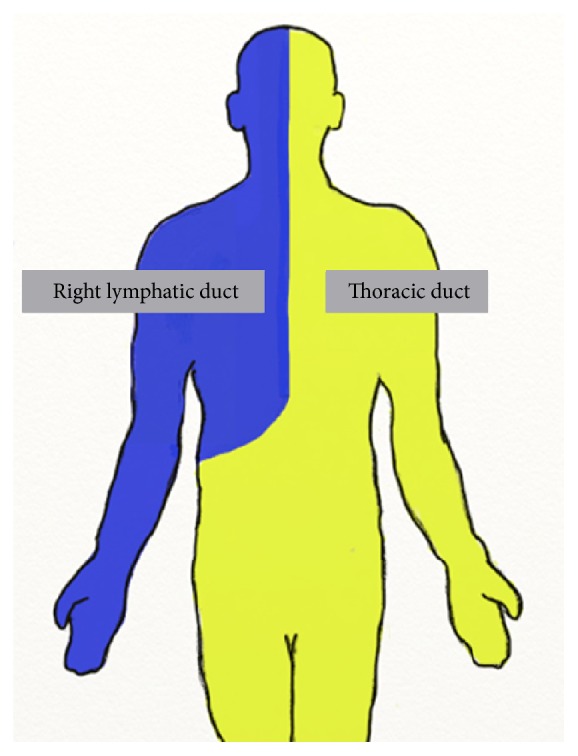
Lymphatic division. The right lymphatic duct collects lymph from the right side of the body, above the diaphragm. The thoracic duct receives lymph from the entire left side of the body and the right side of the body below the diaphragm.

The thoracic duct forms during the 8th week of gestation as two distinct vessels anterior to the aorta, connecting the superior jugular lymph sacs to the inferior cisterna chyli. These vessels develop into the embryonic right and left thoracic ducts and share a number of anastomoses. As the fetus matures, the embryonic thoracic ducts fuse partially to form two distinct lymphatic divisions within the body. The adult thoracic duct is the product of the fusion of the lower 2/3 of the embryonic right duct, the upper 1/3 of the left duct, and their numerous interconnections [9]. The thoracic duct is the largest lymphatic vessel that drains up to 75% of the body's lymph [10] from the entire left body and the right side of the body below the diaphragm [11]. The adult right lymphatic duct receives lymph from the right thorax, arm, and head and neck region (Figure 1). Variations in the course of the thoracic duct are common and may occur as either a persistence of embryonic structures or failure of normal developmental progression. Although unusual, thoracic ducts draining to right internal jugular vein (IJV) have been described [12, 13].

### 2.2. Course of the Thoracic Duct

The thoracic duct originates from the cisterna chyli, a dilated sac at the level of the 2nd lumbar vertebra that receives lymph from intestinal and lumbar lymphatics [14] as well as intercostal lymphatics and periaortic lymph nodes [15]. When the cisterna chyli is congenitally absent, the thoracic duct originates from a haphazard coalescence of lymphatic channels instead [11].

**Figure 2:**
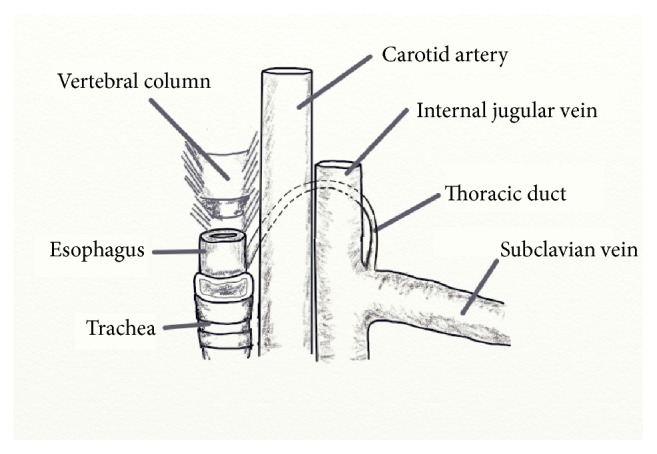
Cervical course of the thoracic duct. The thoracic duct enters the neck lateral to the esophagus, ascending superiorly and laterally behind to the carotid and internal jugular vein before turning inferiorly and anteriorly to join the venous circulation at the confluence of the internal jugular vein and subclavian vein.

Within the abdomen, the thoracic duct ascends along the anterior surface of the lumbar vertebra, between the aorta and azygous vein, to enter the thorax via the aortic hiatus in the posterior mediastinum. Within the thorax, the thoracic duct veers leftward as it continues to ascend, passing posterior to the aortic arch, and enters the root of the left neck lateral to the esophagus. At the root of the neck, the thoracic duct is bordered anteriorly by the left common carotid artery, Vagus nerve, and IJV, medially by the esophagus, laterally by the omohyoid muscle, and posteriorly by the vertebra. From there, the thoracic duct arches superiorly and laterally, anterior to the anterior scalene muscle and phrenic nerve [14]. The thoracic duct generally courses 3–5 cm superior to the clavicle [16]; however it has been reportedly found as high as the level of the superior cornu of the thyroid cartilage [17]. Finally, the thoracic duct turns inferiorly and anteriorly, passing over the subclavian artery to terminate with 1 cm of the confluence of the internal jugular and subclavian veins (Figure 2) [16, 18].

Within this 1 cm region the thoracic duct may terminate into the venous circulation at a number of sites. The most common site is the IJV (46%), followed by the confluence of the IJV and subclavian vein (32%) and the subclavian vein (18%) [11]. Less commonly, the thoracic duct may terminate in the brachiocephalic vein, external jugular vein, or vertebral vein [7, 16].

The thoracic duct generally empties into the venous system as a single duct (76%) [11], though, bifid and trifid configurations have been described [7]. Near its termination, the thoracic duct receives additional lymphatics from subsidiary lymphatic trunks of the left neck (jugular, subclavian, and bronchomediastinal trunks) [16].

The typical length of the adult thoracic duct is 36–45 cm with an average diameter of 5 mm [38]. The diameter of the thoracic duct decreases from the abdomen to the thorax and then increases again in the cervical region, reaching up to 1 cm in diameter as it empties into the venous system [11]. Additionally, rises in intra-abdominal or intrathoracic pressure may further distend the thoracic duct through propagation of hydrostatic forces.

### 2.3. Function of the Thoracic Duct

The thoracic duct is the primary structure that returns lymph from the left body and the right body below the diaphragm to the venous circulation. This includes chyle derived from intestinal lacteals [7, 14]. The thoracic duct serves a crucial role in the maintenance of fluid balance and return of lymph and chyle to the systemic circulation [16].

Chyle is composed of lymphatic fluid and chylomicrons from the gastrointestinal system. Its lymphatic fluid contains protein, white blood cells, electrolytes, fat-soluble vitamins, trace elements, and glucose absorbed from the interstitial fluid, to be returned to the systemic circulation [39]. Chylomicrons consist of esterified monoglycerides and fatty acids combined with cholesterol and proteins. These are formed from the breakdown products of long-chain fatty acids by bile salts and absorbed into the lymphatic system through special lymphatic vessels in the villous region of the intestines known as lacteals. Conversely, the smaller short and medium-chain fatty acids are more water soluble and are absorbed via the intestinal mucosa directly into the hepatic portal vein, thus bypassing the lymphatic system [40].

Chyle is propagated within the thoracic duct primarily by the muscular action of breathing and further facilitated by the duct's smooth muscles and internal valves, which prevent retrograde flow. Factors that modulate chyle flow include diet, intestinal function, physical activity, respiration rate, and changes in intra-abdominal and intrathoracic pressure [40].

## 3. Pathophysiology of Iatrogenic Head and Neck Chyle Leak

### 3.1. Iatrogenic Chylous Fistula in Head and Neck Surgery

Due to its proximity to the IJV and thin vessel wall, the thoracic duct is particularly susceptible to inadvertent injury during dissection low in the neck [8]. Prior irradiation [34] and the presence of metastatic lesions at the confluence of the IJV and subclavian vein [41] make for a more challenging surgical dissection and significantly greater risk of iatrogenic CL.

### 3.2. Chyle Leak Sequelae

Prompt diagnosis and intervention aimed at addressing a CL are essential for favorable surgical outcome. The impact of acute large volume CL includes the loss of protein, fat, and fat-soluble vitamins, trace elements, and lymphocytes in quantities that result in hypovolemia, electrolyte imbalances (hyponatremia, hypochloremia, and hypoproteinemia), malnutrition, and immunosuppression [8, 39, 42, 43].

Wound healing complications can result from the disruption of the normal biochemical milieu, manifesting as delayed wound healing, infection, or wound breakdown with fistula formation. Within the wound bed, extravasated chyle provokes an intense inflammatory reaction, prompting the release of proinflammatory cytokines and tissue proteases that interfere with the healing process. The pressure of accumulated chyle beneath skin flaps may decrease tissue perfusion, resulting in flap necrosis [43]. Systemic metabolic and immunologic derangements associated with CL may further compromise healing [39].

A cervical CL can spread from the root of the neck into the mediastinum. With sufficient hydrostatic pressure, the collection of chyle may penetrate the pleural, forming a chylothorax, which presents clinically with shortness of breath, tachypnea, and chest pain.

## 4. Diagnosis

Chyle leaks may be identified intraoperatively or postoperatively. Due to the potential significant morbidity associated with a CL, leaks identified at the time of surgery should be repaired immediately.

In general, the supraclavicular region should be examined carefully at the conclusion of a head and neck procedure, particularly if the case involves dissection low in the neck. If creamy or milky fluid is noted, the thoracic duct should be identified and ligated [8]. Given the variable course and collapsibility of the thoracic duct and patient fasting in preparation for surgery, identification of the thoracic duct may prove to be difficult. Magnification with surgical loupes or an operative microscope can assist with visualization. Maneuvers that increase intrathoracic or intra-abdominal pressure may facilitate the identification of a CL as well. Trendelenburg positioning and Valsalva maneuver while the anesthesiologist applies positive pressure to raise intrathoracic pressure [16] or manual abdominal compression [44] can propagate hydrostatic forces through the course of the thoracic duct to increase chyle flow and distend the distal thoracic duct to improve visibility. The presence of multiple terminations of the thoracic duct means that even though the thoracic duct may be identified and ligated at the time of surgery, unidentified terminal branches can still result in a CL.

Postoperatively, sudden high increases in drain output, especially following resumption of feedings that contain fat, should raise suspicion of a CL. On examination the neck may exhibit erythema, lymphedema, or a palpable fluid collection in the supraclavicular region. The drain output would have a creamy or milky appearance. A CL can be diagnosed clinically; however, biochemical assay may be helpful for equivocal cases. Drain fluid with triglyceride level greater than 100 mg/dL [45] or serum triglyceride [46, 47] or with the presence of chylomicrons [34] confirms the diagnosis of a CL (Table 1).

## 5. Treatment Options for Chyle Leaks

### 5.1. Intraoperative Chyle Leak

When a CL is identified during surgery, the thoracic duct may be ligated with surgical clips or oversewn with nonabsorbable suture. Additionally, locoregional flaps may be incorporated for additional coverage of the surgical bed. The clavicular head of the sternocleidomastoid can be dissected free and sutured to the wound bed [39]. Although the anterior scalene flap has been described, it is infrequently used due to its small size and the risk of brachial plexus injury during flap harvest [48]. Finally, a rotational pectoralis major flap can provide sufficient tissue bulk and coverage to reliably address a CL [49]. Additional topical agents can be applied to the wound bed at the time of surgery and will be discussed below.

### 5.2. Postoperative Chyle Leak

Following surgery, management of a CL depends on drain output, patient comorbidities, available institutional expertise, and surgeon preference. Chyle leaks may be broadly categorized as low output (<500 mL/day) or high output (>500 mL/day) based on drain output to assist with treatment decision making. In general, low output CL can be treated effectively with conservative management [50], while high output fistulas will often respond unsatisfactorily to conservative management alone and require surgical intervention. With that said, drain output alone should not dictate treatment choices. Treatment effectiveness can often be gauged by how much drain output changes in response to particular interventions.

### 5.3. Conservative Measures

#### 5.3.1. Activity

Because chyle flow is propelled by physical activity, patients with suspected CL should be restricted to bed rest. The head of bed should be elevated (30–40°) [8] and stool softeners provided to reduce intrathoracic and intra-abdominal pressure with bowel movement.

#### 5.3.2. Diet

With potential high volume fluid shift with protein and electrolytes loss, patients with CL need to be monitored for dehydration and malnutrition. Fluid balance and electrolytes should be checked daily and albumin weekly [8]. Intravenous fluids should be administered to achieve euvolemia and electrolytes replenished as needed.

Dietary management plays a crucial role in the nonsurgical management of a CL. All patients with suspected CL should be transitioned to a nonfat diet, low-fat diet, or medium-chain fatty acid (MCFA) diet [51]. In general, a MCFA diet with protein, metabolic mineral mixture, and multivitamin supplementation is preferable to a nonfat diet [52]. Because short- and medium-chain fatty acids are largely water soluble and absorbed via the portal venous circulation rather than the gastrointestinal lymphatics, this special diet bypasses the gastrointestinal lymphatic system, resulting in decreased chyle flow at the CL site, allowing the thoracic duct injury to heal faster. Despite this, a MCFA diet does not stop chyle production entirely.

Orlistat, a pancreatic lipase inhibitor, interferes with lipid metabolism in the duodenum and prevents lipid absorption and may be given as an adjunct to decrease chyle production [53].

Alternatively, patients can be made NPO if the drain output is low and suspected duration of CL is short. NPO is rarely implemented today, as alternative superior dietary options are available that do not contribute to ongoing hypovolemia and malnutrition.

Patients with persistent or high output CL will likely require total parental nutrition (TPN), which bypasses the lymphatic system completely [8, 54]. While more effective than a MCFA diet at reducing chyle production, the use of TPN must be carefully weighed against its need for central venous access, potential complication of increase infection risk, and metabolic disturbances and high cost [55].

#### 5.3.3. Wound Care

The use of pressure dressings remains controversial. Some recommend its use to expedite closure of a CL [6, 8, 50], while others are concerned with its potential compromise of skin flap perfusion [49, 56].

Suction drainage, placed at the time of surgery, is invaluable in the evacuation of extravasated chyle and monitoring of drain output to assess both severity of the CL and treatment effectiveness. While helpful in evacuating high output CL, however, some advocate for the timely removal of suction drainage once its output has diminished sufficiently, to avoid the possibility that the drain suction may prohibit the complete resolution of a CL [5].

Negative wound pressure therapy, or vacuum-assisted closure, with placement of an air-tight seal over the wound and application of negative pressure to the entire wound bed to remove fluid and shrink wound size has had promising results in preliminary reports, but additional studies are needed to test its true effectiveness [51]. Furthermore, negative wound pressure therapy requires exposure of the wound bed.

#### 5.3.4. Somatostatin and Octreotide

Somatostatin is a neuroendocrine hormone discovered in 1973, with numerous effects on the digestive and lymphatic systems [57], and has broad applications for use in therapy for acromegaly, intractable diarrhea, hyperinsulinism, severe gastrointestinal bleeding, pancreatitis, metastatic carcinoids, and tumors secreting vasoactive intestinal peptides [58]. Animal studies in dogs during the early 1980s revealed that intravenous somatostatin significantly reduced thoracic duct lymph flow [59]. Then, building upon this discovery Ulíbarb and colleagues [20] were the first to describe the use of somatostatin for the treatment of CL from thoracic duct injury during a supraglottic laryngectomy in 1990.

Somatostatin decreases chyle production via reduction of gastric, pancreatic, and intestinal secretions [36, 51, 60]. It constricts smooth muscles in splanchnic and lymphatic vessels to decrease lymph production [51] and lymph flow [42], respectively.

Somatostatin's major drawback is its short half-life, which requires continuous intravenous infusion. This problem was solved with the development of octreotide, somatostatin's long-acting analog, which permitted administration with long-lasting subcutaneous injections [34]. Octreotide has gained considerable popularity in the management of CL, first in thoracic surgery and more recently with head and neck surgery. Octreotide is a cost-effective therapy for iatrogenic CL that significantly decreases morbidity, length of stay, and need for surgical intervention [34].

From 2001 to 2015 seventeen studies investigating the effectiveness of octreotide in the management of cervical CL were published (Table 2). With the exception of two large case series, most publications were case reports. Jain et al. [24] recounted their experience treating CL in 19 left modified radical neck dissection patients and Swanson et al. [34] shared their results treating CL in 12 patients undergoing a number of different head and neck procedures. In these studies, total of 49 patients were treated with subcutaneous octreotide for their CL. Surgeries cited included thyroidectomy with or without neck dissection, modified radical and radical neck dissection, and parathyroidectomy. Chyle leaks occurred with both left and right neck dissections. Nearly all of the studies cited use of suction drainage and dietary modifications. Less than one-third of the authors applied pressure dressings.

To date, there are no consensus guidelines on the optimal octreotide treatment dose and duration in CL management. In our literature review, the decision of what dosage to use was often anecdotal and occasionally increased by some of the authors when perceived ineffective. Octreotide dosage ranged from 100 *μ*g subcutaneous every 8 to 12 hours to 200 *μ*g subcutaneous every 8 hours [35]. Time from initiation of octreotide therapy to CL cessation ranged from 1 to 15 days, and total octreotide treatment duration varied widely from 3 to 24 days. In general, octreotide was administered an additional 1-2 days after CL cessation to ensure complete resolution. In Jain et al.'s [24] study, low output leaks (<500 mL/day) stopped after 2–4 days of octreotide and these patients were given a total of 5 days of octreotide; high output leaks (>500 mL/day) resolved after 5 days of octreotide and this cohort was treated for a total of 7 days. Although Swanson et al. [34] did not stratify their treatment groups by drain output, CL in their series resolved on average 5.5 days after initiation of octreotide therapy, and their patients were treated for approximately 9 days total.

The most commonly associated side effects of octreotide are nausea, abdominal discomfort, and diarrhea. Rare but serious complications include hypoglycemia and cholecystitis secondary to cholestasis [61]. In less than 1% of patients, anaphylactic shock, gastrointestinal bleeding, and pulmonary embolism have been described. Octreotide should be prescribed with caution in patients with preexisting cardiovascular and hepatic disease [34]. Most adverse effects are dose and duration dependent [27].

Octreotide has emerged as a powerful adjunct in the conservative management of CL and should be a part of the armamentarium of every head and neck surgeon. However, not every CL will respond completely to octreotide therapy alone. In our literature review, two patients required surgical reexploration for control of their CL, despite a trial of octreotide [23, 25]. Most authors agreed that if there is no reduction in drain output after 5 days of octreotide therapy then surgical exploration is indicated [39].

### 5.4. Topical Agents

Sclerosing agents such as OK-432 or tetracycline administered at the time of surgery or postoperatively through drainage tubing or percutaneous injection can generate fibrosis to seal a CL [3, 39]. Should the CL persist, however, the surgical field obliteration by the sclerosing agent makes reoperation considerably more challenging. Furthermore, sclerosing agents should be used with care, as it could potentially injure surrounding structures in the wound bed. Phrenic nerve paralysis after doxycycline sclerotherapy for CL has been reported [62].

Cyanoacrylate adhesives, fibrin glue [63–65], and polyglactin (Vicryl) mesh [64] have been placed at the time of surgery, with success, for controlling visible CL.

### 5.5. Surgical Exploration

Surgical reexploration should be considered only after conservative measures have either been exhausted or deemed ineffective. Suggested criteria for reexploration range from outputs of >500 mL/day to >1000 mL/day output for 5 days [1, 8, 22, 66]. Although the recommended criteria for reexploration vary considerably, the general sense is that surgical reexploration should take place when a CL does not respond appropriately to conservative management. Generally speaking, surgical intervention should be decided upon within first 4-5 days of a CL, when prompt response to medical management is absent [6].

At the time of reexploration, local inflammation from extravasated chyle can make thoracic duct identification difficult. Trendelenberg positioning and maneuvers that raise intrathoracic and intra-abdominal pressure can facilitate identification of the site of the CL. Having the patient ingest a fatty diet before surgery can stimulate chyle production and aid in CL localization as well.

As described above, when identified, the leaking thoracic duct can be ligated, covered with a muscle flap, or treated with any number of sclerosing agents, adhesive agents, or mesh. It is imperative that a suction drain is placed at the conclusion of the case.

### 5.6. Distant Management

In certain instances, when there is a persistent CL after surgical reexploration or when reexploration may not be ideal because of distorted anatomy or tenuous in the case of a microvascular free flap, the head and neck surgeon may seek the assistance of his interventional radiology or thoracic-foregut colleagues for distant management of a thoracic duct leak.

Percutaneous transabdominal cannulation of the thoracic duct at the cisterna chyli with lymphography and selective distal embolization with coils or tissue adhesive is a safe and minimally invasive technique for the treatment of CL that do not respond to conservative management, with a reported success rate of 45–70% [45, 51]. Given the relative low morbidity and reasonable success rate, this may be a viable alternative to surgical exploration, if one's facility has the appropriate equipment and personnel. The major drawback to this method is that it can be time-consuming and often require multiple attempts [67].

For patients with failed surgical ligation, thoracoscopic ligation can be an effective salvage procedure that addresses the thoracic duct proximally [39]. Exposure and ligation of the thoracic duct are performed through a right sided thoracoscopic approach, through which the thoracic duct is ligated at the supradiaphragmatic hiatus between the aorta and azygous vein [66, 68].

## 6. Discussion

The variable course and fragile composition of the thoracic duct make it vulnerable to iatrogenic injury during head and neck procedures that involve dissection low in the neck. In certain instances, inadvertent injury to the thoracic duct is unavoidable, particularly with the extirpation of malignancy. Fear of iatrogenic CL should not preclude sound oncologic resection. Rather, identification and ligation of a CL during surgery or its timely recognition and treatment in the postoperative period are essential for best surgical outcomes.

An appreciation of the anatomy, variable course, and possible termination patterns of the thoracic duct will lead to a more comprehensive management of any potential intraoperative CL. Additionally, increases in intrathoracic and intra-abdominal pressure and preoperative feeding of a fatty meal can help with localization of a CL.

The surgical care team should be vigilant for a CL if the surgery involved dissection in the vicinity of the confluence of the IJV and subclavian vein, on either side of the neck. High drain output, sudden increase of drain output after resumption of enteral feeds, or a creamy appearance of the drain output should all raise suspicion of a CL. A CL can be diagnosed clinically; however, in ambiguous cases, biochemical assay of drain contents may be helpful.

Chyle leaks can significantly impact wound healing and cause hypovolemia, malnutrition, electrolyte disturbances, and immunosuppression. Therefore, conservative management should be initiated immediately when a CL is diagnosed following surgery. This includes bed rest and head of bed elevation with a MCFA/nonfat diet or TPN. Fluid balance, electrolytes, and protein status should also be monitored closely.

**Figure 3:**
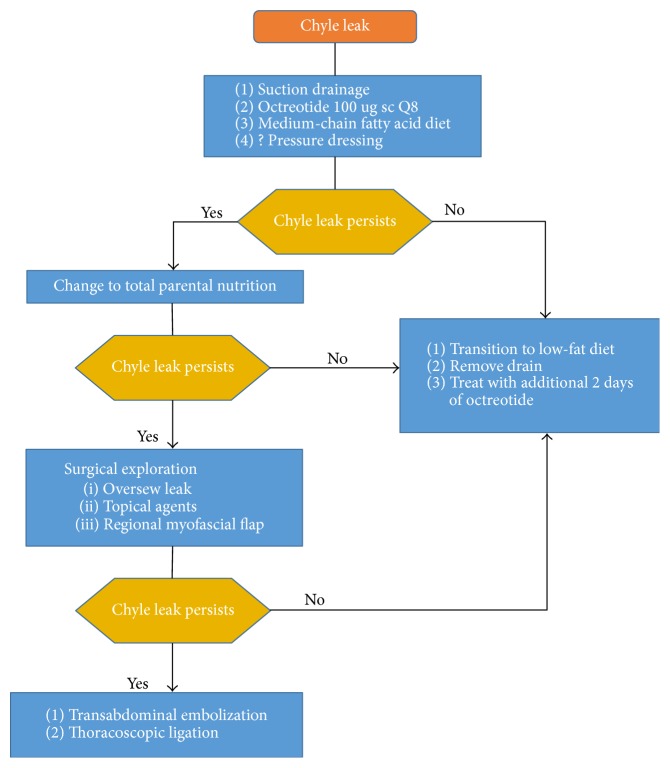
Proposed treatment algorithm for the postoperative chyle leak.

If a CL does not respond satisfactorily to conservative management alone, surgical control locally or distantly should be considered. There is much debate about the exact criteria for and timing of surgical reexploration. Muscle flaps, sclerosing agents, and adhesives can be applied at the time of surgery as an adjunct to thoracic duct ligation. Suction drainage is essential for evacuation of chyle from the wound bed and to monitor output. For recalcitrant CL or circumstances that preclude reexploration, CL can be addressed distantly with thoracic duct catheterization and embolization or thoracoscopic thoracic duct ligation. The services available at each medical institution may differ and should be taken into account when deciding on the best management plan (Figure 3).

## 7. Conclusion

Chyle leak formation is an uncommon but serious sequela of head and neck surgery, particularly with the resection of malignancy low in the neck. Chyle extravasation can result in delayed wound healing, dehydration, malnutrition, electrolyte disturbances, and immunosuppression. Prompt identification and treatment of a chyle leak are essential for optimal surgical outcomes.

## Figures and Tables

**Table 1:** Diagnosing a chyle leak.

Clinical	Drain output biochemical assay
(i) Sudden increase in drain output, especially immediately following enteral feeding (ii) Supraclavicular erythema, lymphedema, or palpable fluid collection (iii) Creamy or milky drain output	(i) Triglyceride > 100 mg/dL (ii) Triglyceride > serum triglyceride level (iii) Presence of chylomicrons

**Table 2:** Literature review of somatostatin and octreotide for treatment of chyle leak.

Author	Year	Patients	Surgery	Treatment dosage	Treatment duration	Treatment start to leak cessation	Additional measures	Comments
Somatostatin								

Coşkun and Yildirim [19]	2010	1	Thyroidectomy + L MRND	3 mg iv Qday	5 days	1 day	Suction drainage Dietary modifications	

Ulíbarb et al. [20]	1990	1	Supraglottic laryngectomy + L MRND	6 mg iv Qday	12 days	5 days	Suction drainage Dietary modifications Pressure dressing	

Octreotide								

Ahn et al. [21]	2012	2	L MRND (1) Thyroidectomy + R MRND (1)	100 *µ*g sc Q8–12	11 days	11 days	Suction drainage Pressure dressing	

Al-Sebeih et al. [22]	2001	1	Total laryngectomy + B MRND	100 *µ*g sc Q8	Not specified	3 days	Suction drainage Dietary modifications	B chylothoraces requiring chest tubes

Harlak et al. [23]	2008	1	R MRND	100 *µ*g sc Q8	15 days	15 days	Suction drainage Dietary modifications Pressure dressing Surgical exploration w/fibrin glue	Metastatic breast cancer Reexploration for persistent leak

Jain et al. [24]	2015	19	Left neck dissection (19)	100 *µ*g sc Q8	Low output 5 days High output 7 days	Low output 2–4 days High output 5 days	Suction drainage Dietary modifications Pressure dressing	Low output <500 mL/24 hours, high output >500 mL/24 hours

Jiménez et al. [25]	2008	1	Thyroidectomy + B MRND	100 *µ*g sc Q8 > somatostatin 6 mg iv Qday	11 days	N/A	Suction drainage Dietary modifications Surgical Exploration	Right sided chyle leak Leak resolved with definitive surgical intervention Switched from octreotide to somatostatin

El Dabe Mikhail et al. [26]	2009	1	Thyroidectomy	Not specified	5 days	5 days	Dietary modifications Pressure dressing	

Nyquist et al. [27]	2003	1	Thyroidectomy + L MRND	100 *µ*g sc Q8	8 days	1 day	Suction drainage Dietary modifications Pressure dressing	

Ogi et al. [28]	2013	1	Thyroidectomy + B MRND	100 *µ*g sc Q12	3 days	3 days	Suction drainage Dietary modifications	

Prabhu and Thomas [29]	2015	1	L radical neck dissection	100 *µ*g sc Q8	14 days	14 days	Suction drainage Dietary modifications Surgical exploration w/tetracycline	L chylothorax Side effect: vomiting

Rodier et al. [30]	2011	1	Thyroidectomy + central & L MRND (1)	100 *µ*g sc Q8	6 days	6 days	Suction drainage Dietary modifications	

Srikumar et al. [31]	2006	1	L radical neck dissection	200 *µ*g sc Qday	14 days	14 days	Suction drainage Dietary modifications	B chylothoraces requiring chest tubes

Suver et al. [32]	2004	1	B MRND + mediastinal dissection	4 *µ*g/kg/hr	7	7	Suction drainage Dietary modifications	Lymphatic malformation in 10-month-old child

Suslu et al. [33]	2014	3	Thyroidectomy + L MRND (1) Thyroidectomy + B MRND (1) Radical neck dissection (1)	100 *µ*g sc Q8	7.5	6.5	Suction drainage Dietary modifications Surgical exploration	L chylothorax (1) Failed surgical exploration (2) Laterality not specified

Swanson et al. [34]	2015	12	L MRND (3) R MRND (1) Thyroidectomy + B MRND (3) Thyroidectomy (4) Parathyroidectomy (1)	100 *µ*g sc Q8	9.4	5.5	Suction drainage Dietary modifications	

Touska et al. [35]	2002	1	R completion thyroid lobectomy	200 *µ*g sc Q8	17	10	Suction drainage Dietary modifications	No suction drain initially Reoperation for suspected abscess Discharged with 7-day course of octreotide

Valentine et al. [36]	2002	1	Thyroidectomy + L MRND	50–100 *µ*g sc Q8	24	24	Suction drainage Dietary modifications	Octreotide dose increased from 50 ug to 100 *µ*g sc Q8 not effective

Khurana et al. [37]	2009	1	Thyroidectomy + B MRND	100 *µ*g sc Q8	Not specified	Not specified	Suction drainage Dietary modifications Surgical exploration	B chylothoraces requiring chest tubes Leak site not identified

L = left; R = right; B = bilateral; MRND = modified radical neck dissection.

## Abbreviations

CL: Chyle leak
TPN: Total parental nutrition
IJV: Internal jugular vein
MCFA: Middle chain fatty acid.
